# Supplementary material for: A Deep Gravity model for mobility flows generation
Source: Nat Commun. 2021 Nov 12;12:6576. doi: 10.1038/s41467-021-26752-4 (PMC8589995; doi:10.1038/s41467-021-26752-4)
Supplement: Supplementary file 1 — Supplementary Information [file 41467_2021_26752_MOESM1_ESM.pdf]

A Deep Gravity model for mobility flows generation

Supplementary Material

Filippo Simini, Gianni Barlacchi, Massimiliano Luca, Luca Pappalardo

1 Supplementary Figures

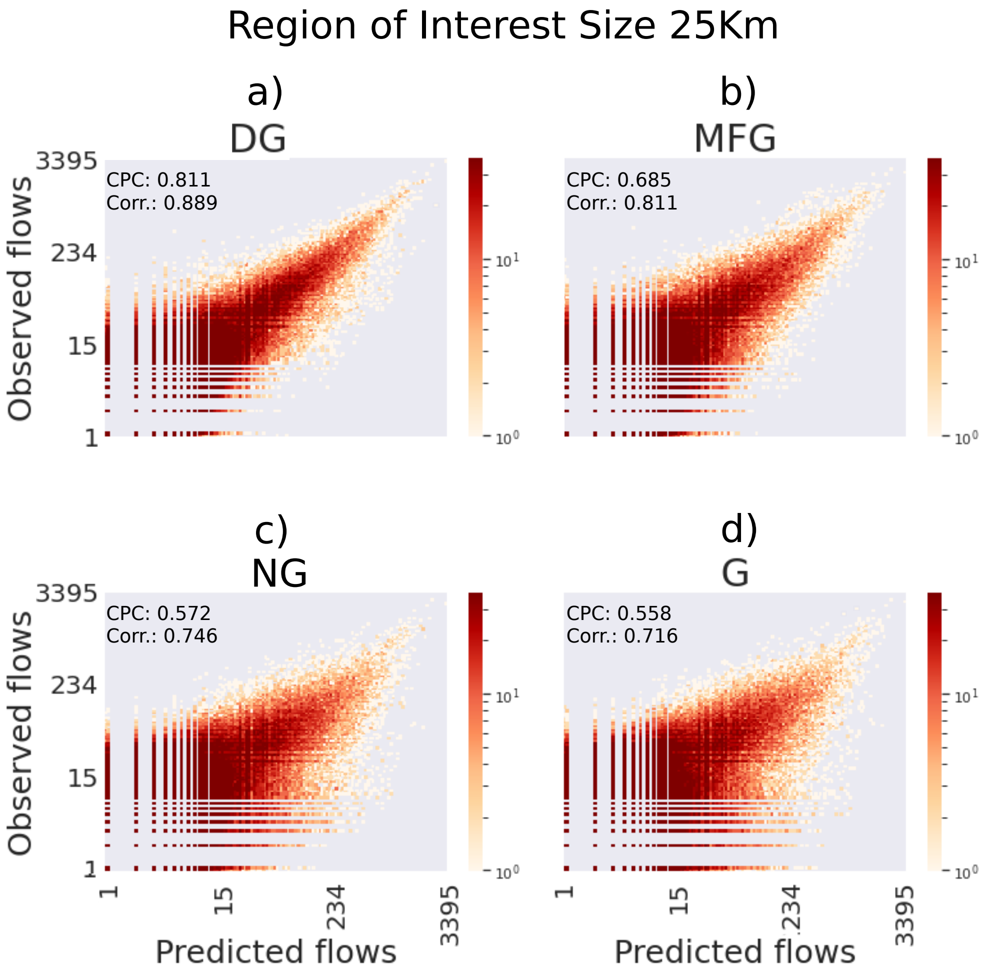

Supplementary Figure 1: Observed flows versus predicted flows between MSOAs within regions of interest of 25km for DG (a), MFG (b), NG (c) and G (d). The color, in a gradient from yellow to red, indicates the density of points (flows). For each plot, we specify the CPC and the Pearson correlation between the observed and the predicted flows. MSOAs (Middle Layer Super Output Areas) are an aggregation of adjacent OAs with similar social characteristics; they generally contain 5,000 to 15,000 residents and 2,000 to 6,000 households. Plots with a higher concentration of points closer to the main diagonal have a higher correlation and a higher CPC.

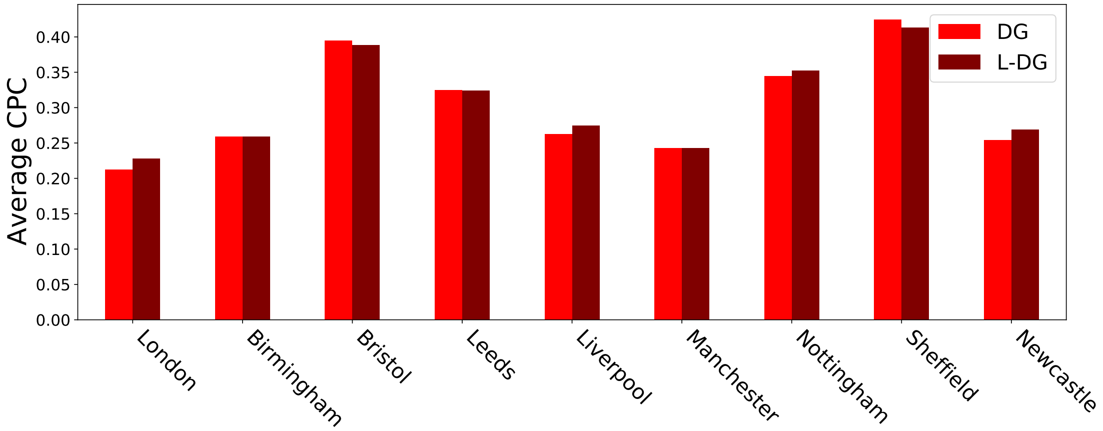

Supplementary Figure 2: Average CPC of DG and L-DG (Leave-One-City-Out DG) on the Core Cities and London according to the leave-one-city-out validation.

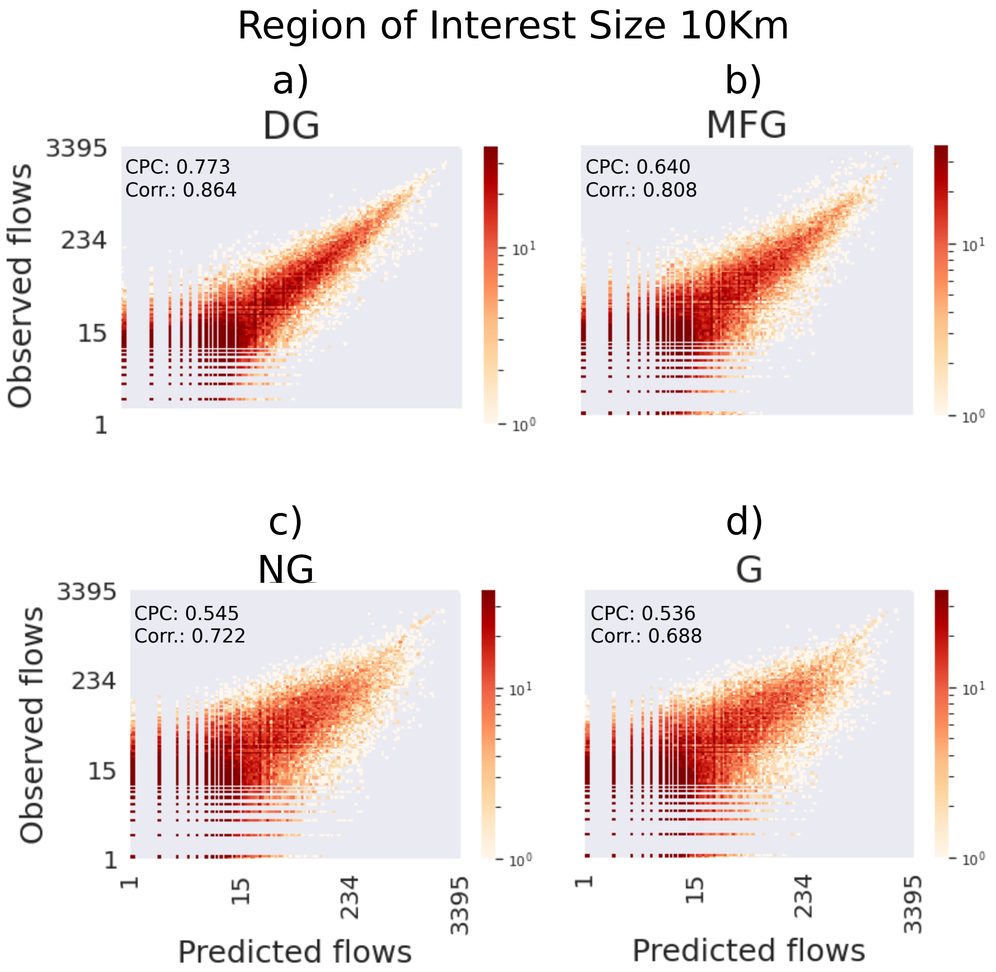

Supplementary Figure 3: Observed flows versus predicted flows between MSOAs within regions of interest of 10km for DG (a), MFG (b), NG (c) and G (d). The color, in a gradient from yellow to red, indicates the density of points (flows). For each plot, we specify the CPC and the Pearson correlation between the observed and the predicted flows. MSOAs (Middle Layer Super Output Areas) are an aggregation of adjacent OAs with similar social characteristics; they generally contain 5,000 to 15,000 residents and 2,000 to 6,000 households. Plots with a higher concentration of points closer to the main diagonal have a higher correlation and a higher CPC.

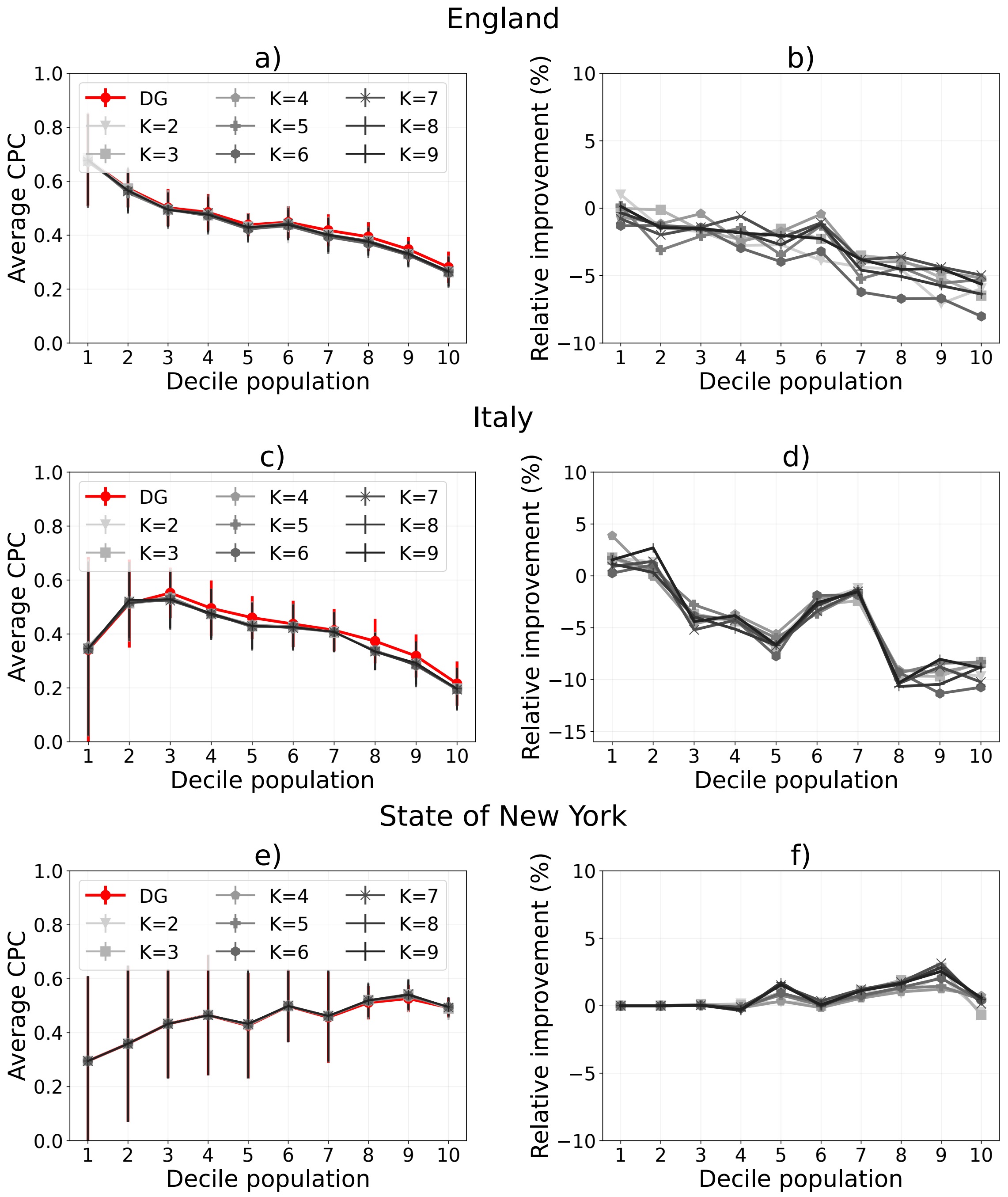
England
a)
b)
Italy
c)
d)
State of New York
e)
f)
DG
K=2
K=3
K=4
K=5
K=6
K=7
K=8
K=9
Average CPC
Relative improvement (%)
Decile population

Supplementary Figure 4: **Performances of DG-Knn.** (a,c,e) Comparison of the performance of DG and DG-Knn (for $k = 2, \ldots, 9$) in terms of Common Part of Commuters (CPC), varying the decile of the population and for regions of interest sizes of 25km for England (a), Italy (c) and New York State (e). Markers indicate the average CPC for a decile. Error bars indicate the standard deviation of CPC of each decile. We do not find any significant improvement in the performance of the model as k increases, regardless of the decile of the population. (b,d,f) Relative improvement of DG-Knn (for $k = 2, \ldots, 9$) with respect to G, varying the decile of the population and for region of interest of sizes 25km for England (b), Italy (d) and New York State (f). We run five independent experiments in which we train the models on 50% of randomly chosen tiles, stratifying according to the population in the decile, corresponding to 84,491.63 Output Areas for England, 200,746.15 Census Areas for Italy and 2118.93 Census Tracts for New York State. The remaining 50% of the tiles are used to evaluate the performance of the models in terms of CPC. The improvement of DG-Knn with respect to G is negligible and, for high deciles, even negative.

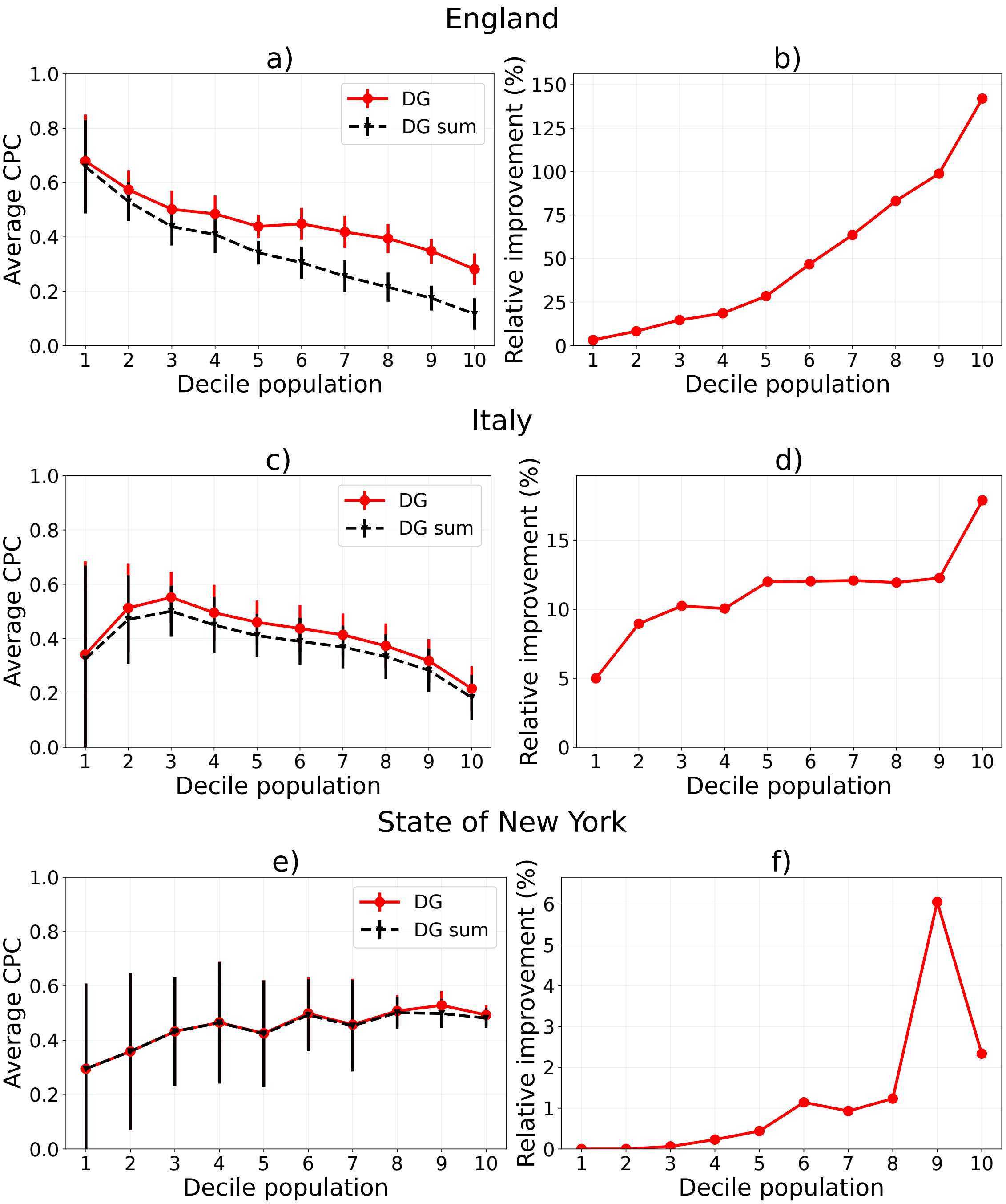
England
a)
b)
Average CPC
Relative improvement (%)
Decile population
DG
DG sum
Italy
c)
d)
State of New York
e)
f)

Supplementary Figure 5: **Performances of DG-sum.** (a,c,e) Comparison of the performance of DG and DG-sum in terms of Common Part of Commuters (CPC), varying the decile of the population and for regions of interest sizes of 25km for England (a), Italy (c) and New York State (e). (b,d,f) Relative improvement of DG with respect to DG-sum, varying the decile of the population and for region of interest of sizes 25km for England (b), Italy (d) and New York State (f). We run five independent experiments in which we train the models on 50% of randomly chosen tiles, stratifying according to the population in the decile, corresponding to 84,491.63 Output Areas for England, 200,746.15 Census Areas for Italy and 2118.93 Census Tracts for New York State. The remaining 50% of the tiles are used to evaluate the performance of the models in terms of CPC.

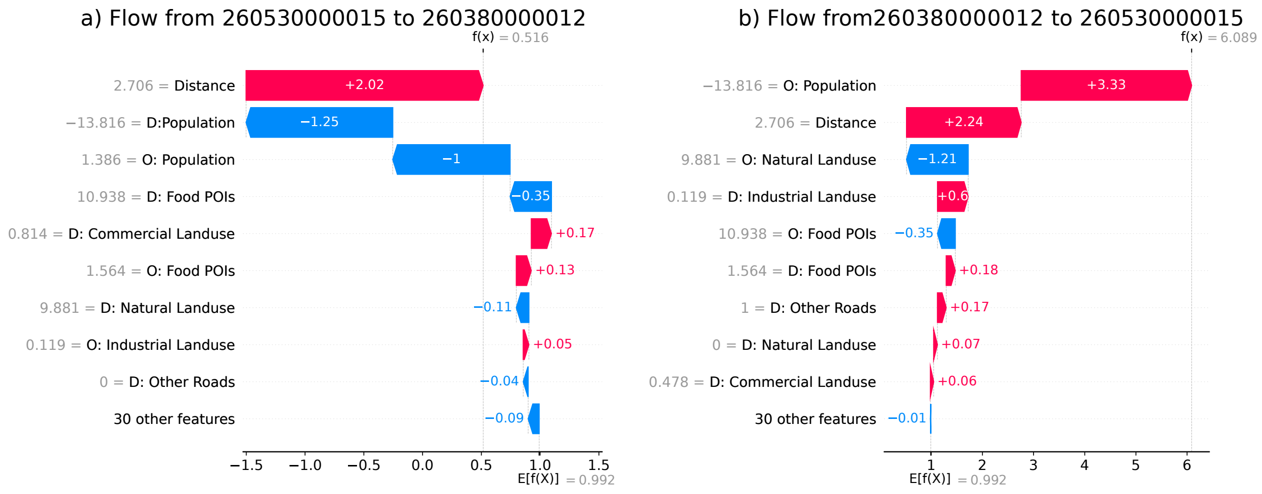

Supplementary Figure 6: **Explaining generated flows in Italy.** (a, b) Shapely values for the two flows between census area 260380000012 (population of about 501 individuals) and census area 260530000015 (population of about 131 individuals).

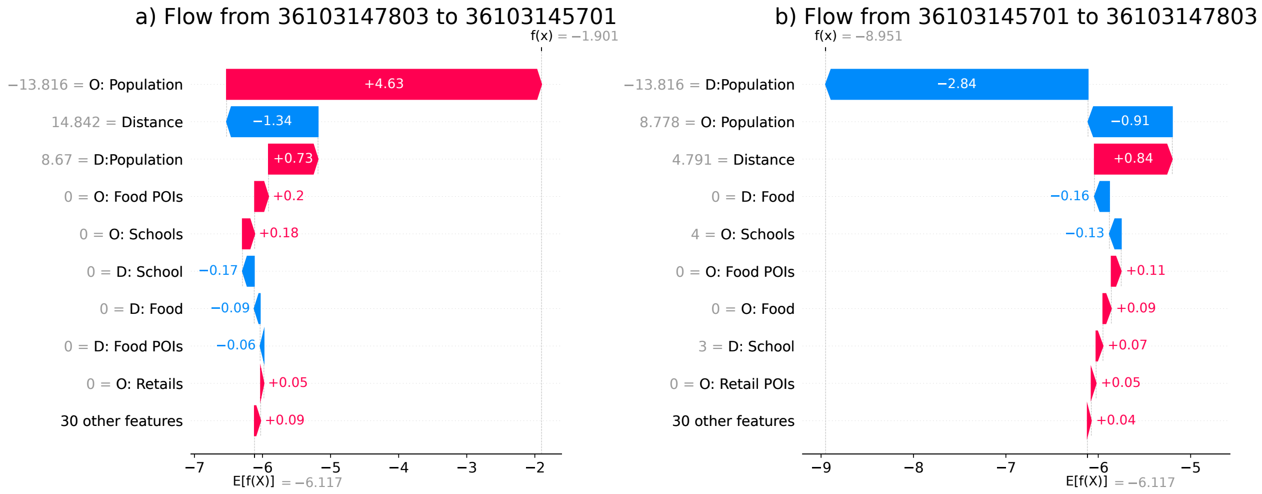

Supplementary Figure 7: **Explaining generated flows in New York.** (a, b) Shapely values for the two flows between census tract 36103147803 (population of about 6487 individuals) and census tract 36103145701 (population of about 11,757 individuals).

2 Supplementary Tables

Country/State	Aggregation Unit	Number of Tiles	Number of Units	Avg Units per Tile	Avg Units Area (km^2)
England	Output Areas (OAs)	885 (25 km), 3669 (10 km)	173,320	195.84 (25 km), 46.88 (10 km)	1.069
Italy	Census Areas (CAs)	1551 (25 km), 6154 (10 km)	402,678	259.62 (25 km), 65.43 (10 km)	0.751
New York	Census Tracts (CTs)	475 (25 km), 1082 (10 km)	5367	11.29 (25 km), 4.96 (10 km)	45.057

Table 1: Statistics about the datasets. The spatial unit adopted is the smallest available (i.e., the most fine-grained). In England, we use the Output Areas (OAs) provided by the UK census 2011. We create a tessellation of England into 885 squares of 25×25 km^2 (with 195.84 OAs per tile on average) and into 3669 squares of 10×10 km^2 (with 46.88 OAs per tile on average). Similarly, in Italy, we use the Census Areas (CAs) provided by the Italian national census bureau (ISTAT) and map them with 1551 squared tiles of 25×25 km^2 (259.62 CAs per tile on average) and 6154 tiles of 10×10 km^2 (65.43 CAs per tile on average). In US, we use Census Tracts (CTs), which are bigger that those of Italy and England. For this reason, we have fewer areas (5367 CTs in New York State) and on average 11.29 CTs per tile (25×25 km^2) and 4.96 CTs per tile (10×10 km^2).

		Decile of Population										Global Metrics			
		1	2	3	4	5	6	7	8	9	10	CPC	NRMSE	Corr.	JSD
England (25 Km)															
G	Mean CPC	0.66	0.51	0.40	0.34	0.28	0.25	0.20	0.16	0.12	0.08	0.11	0.51	0.35	0.73
	std CPC	0.18	0.09	0.07	0.04	0.04	0.03	0.03	0.02	0.02	0.02				
NG	Mean CPC	0.64	0.50	0.41	0.36	0.31	0.27	0.21	0.16	0.13	0.08	0.12	0.45	0.56	0.72
	std CPC	0.18	0.07	0.06	0.07	0.06	0.04	0.03	0.02	0.02	0.02				
	Rel. Imp.	-1.52	-1.88	0.35	5.79	6.41	4.41	3.82	4.46	3.99	4.53				
MFG	Mean CPC	0.66	0.52	0.45	0.41	0.36	0.36	0.32	0.30	0.26	0.19	0.23	0.47	0.48	0.65
	std CPC	0.17	0.09	0.07	0.05	0.04	0.04	0.06	0.05	0.04	0.04				
	Rel. Imp.	1.29	1.55	13.46	20.11	26.89	43.01	61.43	87.83	105.64	139.46				
**DG**	Mean CPC	**0.67**	**0.57**	**0.50**	**0.48**	**0.44**	**0.45**	**41**	**0.39**	**0.35**	**0.28**	**0.32**	**0.41**	**0.61**	**0.60**
	std CPC	0.17	0.07	0.06	0.06	0.04	0.05	0.05	0.05	0.04	0.05				
	Rel. Imp.	**3.20**	**11.72**	**24.91**	**41.47**	**54.35**	**75.76**	**108.47**	**143.54**	**174.97**	**246.88**				
England (10 Km)															
G	Mean CPC	0.80	0.72	0.64	0.55	0.48	0.41	0.34	0.27	0.20	0.12	0.15	0.66	0.44	0.70
	std CPC	0.30	0.11	0.09	0.09	0.07	0.07	0.06	0.05	0.05	0.04				
NG	Mean CPC	0.81	0.73	0.65	0.56	0.50	0.42	0.36	0.29	0.21	0.13	0.17	0.69	0.61	0.69
	std CPC	0.31	0.12	0.11	0.09	0.09	0.07	0.07	0.06	0.05	0.04				
	Rel. Imp.	0.73	1.20	1.11	2.07	3.86	3.54	5.85	7.61	6.89	9.82				
MFG	Mean CPC	0.81	0.72	0.65	0.57	0.52	0.46	0.42	0.38	0.32	0.26	0.28	0.84	0.41	0.62
	std CPC	0.30	0.11	0.08	0.08	0.07	0.07	0.08	0.07	0.08	0.07				
	Rel. Imp.	0.11	0.28	1.69	3.87	6.96	12.45	22.66	38.39	62.12	114.03				
**DG**	Mean CPC	**0.81**	**0.74**	**0.67**	**0.60**	**0.57**	**0.52**	**0.50**	**0.46**	**0.41**	**0.34**	**0.36**	**0.66**	**0.65**	**0.57**
	std CPC	0.29	0.11	0.09	0.09	0.08	0.07	0.08	0.07	0.07	0.08				
	Rel. Imp.	**0.59**	**2.25**	**3.96**	**9.08**	**16.74**	**26.97**	**43.31**	**67.01**	**103.41**	**177.82**				
Italy (25 Km)															
G	Mean CPC	0.26	0.38	0.41	0.37	0.31	0.29	0.27	0.24	0.21	0.13	0.18	0.48	0.49	0.69
	std CPC	0.27	0.14	0.09	0.09	0.08	0.07	0.06	0.06	0.05	0.05				
NG	Mean CPC	0.31	0.44	0.48	0.43	0.38	0.35	0.34	0.30	0.25	0.15	0.21	0.45	0.57	0.67
	std CPC	0.31	0.14	0.10	0.10	0.08	0.07	0.06	0.07	0.06	0.06				
	Rel. Imp.	19.30	14.63	16.41	16.82	19.76	22.26	23.67	22.93	19.93	19.45				
MFG	Mean CPC	0.29	0.41	0.45	0.41	0.37	0.33	0.31	0.28	0.23	0.14	0.20	0.50	0.44	0.67
	std CPC	0.30	0.16	0.09	0.09	0.09	0.07	0.06	0.07	0.06	0.06				
	Rel. Imp.	10.96	5.95	10.68	10.88	15.62	14.76	14.69	15.70	13.99	14.20				
**DG**	Mean CPC	**0.34**	**51**	**0.55**	**0.49**	**0.46**	**0.43**	**0.41**	**0.37**	**0.31**	**0.21**	**0.27**	**0.43**	**0.62**	**0.63**
	std CPC	0.34	0.16	0.09	0.10	0.07	0.08	0.07	0.08	0.07	0.08				
	Rel. Imp.	**30.02**	**31.62**	**32.98**	**33.26**	**43.97**	**48.46**	**49.18**	**52.35**	**51.46**	**66.02**				
Italy (10 Km)															
G	Mean CPC	0.25	0.35	0.39	0.37	0.34	0.29	0.28	0.25	0.20	0.12	0.18	0.49	49	0.69
	std CPC	0.27	0.14	0.11	0.09	0.08	0.07	0.05	0.06	0.06	0.05				
NG	Mean CPC	0.28	0.41	0.47	0.44	0.41	0.36	0.36	0.32	0.25	0.16	0.23	0.46	0.58	0.66
	std CPC	0.32	0.15	0.12	0.10	0.08	0.09	0.05	0.07	0.08	0.07				
	Rel. Imp.	14.98	17.23	19.99	18.01	20.70	25.31	29.46	25.80	25.95	29.56				
MFG	Mean CPC	0.29	0.39	0.43	0.41	0.39	0.33	0.32	0.29	0.22	0.14	0.21	0.61	0.33	0.67
	std CPC	0.31	0.16	0.12	0.09	0.08	0.09	0.05	0.06	0.07	0.06				
	Rel. Imp.	16.34	11.88	10.04	9.98	13.05	14.97	15.03	15.26	13.77	15.89				
**DG**	Mean CPC	**0.31**	**0.48**	**0.52**	**0.48**	**0.47**	**0.42**	**0.42**	**0.38**	**0.30**	**0.21**	**0.28**	**0.43**	**0.64**	**0.63**
	std CPC	0.35	0.18	0.12	0.09	0.08	0.10	0.05	0.07	0.08	0.08				
	Rel. Imp.	**25.33**	**35.77**	**31.61**	**29.84**	**35.43**	**43.45**	**49.98**	**48.32**	**53.84**	**68.07**				

		Decile of Population										Global Metrics			
		1	2	3	4	5	6	7	8	9	10	CPC	NRMSE	Corr.	JSD
New York State (25 Km)															
G	Mean CPC	0.29	0.35	0.24	0.25	0.13	0.13	0.16	0.09	0.06	0.04	0.06	0.74	0.03	0.78
	std CPC	0.31	0.28	0.25	0.26	0.19	0.18	0.19	0.14	0.04	0.02				
NG	Mean CPC	0.29	0.35	0.43	0.46	0.42	0.49	0.45	0.50	0.49	0.47	0.68	0.26	0.93	0.34
	std CPC	0.31	0.28	0.20	0.22	0.19	0.13	0.16	0.05	0.02	0.03				
	Rel. Imp.	0	0	75.71	81.90	206.35	278.09	180.91	405.66	607.17	1031.14				
MFG	Mean CPC	0.29	0.35	0.36	0.37	0.31	0.33	0.28	0.28	0.27	0.18	0.28	0.48	0.35	0.62
	std CPC	0.31	0.28	0.18	0.19	0.15	0.10	0.13	0.07	0.08	0.03				
	Rel. Imp.	0	0	49.70	48.08	126.68	157.01	74.47	184.04	297.44	351.59				
**DG**	Mean CPC	**0.29**	**0.35**	**0.43**	**0.46**	**0.42**	**0.49**	**0.45**	**0.51**	**0.52**	**0.49**	**0.70**	**0.19**	**0.93**	**0.33**
	std CPC	0.31	0.28	0.20	0.22	0.19	0.13	0.16	0.06	0.05	0.03				
	Rel. Imp.	**0**	**0**	**75.80**	**82.41**	**208.03**	**282.91**	**184.33**	**416.43**	**661.03**	**1076.93**				
New York State (10 Km)															
G	Mean CPC	0.28	0.35	0.34	0.38	0.37	0.32	0.21	0.20	0.14	0.09	0.22	0.56	0.25	0.68
	std CPC	0.26	0.26	0.24	0.21	0.23	0.22	0.20	0.19	0.11	0.03				
NG	Mean CPC	0.28	0.35	0.34	0.38	0.37	0.32	0.36	0.40	0.44	0.45	0.78	0.18	0.97	0.27
	std CPC	0.26	0.26	0.24	0.21	0.23	0.22	0.19	0.17	0.11	0.03				
	Rel. Imp.	0	0	0	0	0	0	69.52	98.56	213.73	359.18				
MFG	Mean CPC	0.28	0.35	0.34	0.38	0.37	0.32	0.30	0.29	0.24	17	0.34	0.39	0.48	0.58
	std CPC	0.26	0.26	0.24	0.21	0.23	0.22	0.17	0.15	0.09	0.05				
	Rel. Imp.	0	0	0	0	0	0	38.36	44.82	73.95	81.22				
**DG**	Mean CPC	**0.28**	**0.35**	**0.34**	**0.38**	**0.37**	**0.32**	**0.36**	**0.40**	**0.45**	**0.46**	**0.79**	**0.17**	**0.97**	**0.26**
	std CPC	0.26	0.26	0.24	0.21	0.23	0.22	0.19	0.17	0.12	0.04				
	Rel. Imp.	**0**	**0**	**0**	**0**	**0**	**0**	**70.28**	**100.16**	**224.65**	**374.11**				

Table 2: **Experimental results.** Comparison of the performance, in terms of Common Part of Commuters (CPC), of Gravity (G), Nonlinear Gravity (NG), Multi-Feature Gravity (MFG), and Deep Gravity (DG), varying the decile of the population of the regions of interest. For each model, and for each decile of the distribution of population, we show the average CPC and the standard deviation of the CPC obtained over five runs of the model. For NG, MFG, and DG we also show the relative improvement in terms of CPC with respect to model G. We put in bold the values over the deciles that correspond to the best mean CPC and relative improvement.

3 Supplementary Notes

Supplementary Note 1: evaluation metrics. The Pearson Correlation coefficient measures the linear dependence between two variables (sets of flows) and it is defined as:

$$r = \frac{\sum_{t=1}^{n}(y_i - \bar{y})(\hat{y}_i - \bar{\hat{y}})}{\sqrt{\sum_{i=1}^{n}(y_i - \bar{y})^2}\sqrt{\sum_{i=1}^{n}(\hat{y}_i - \bar{\hat{y}})^2}} \,. \tag{1}$$

where n is the sample size (the number of flows), $\hat{y}_i$ indicates the generated flow, y_i indicates the actual flow, and $\bar{y}$ and $\bar{\hat{y}}$ indicate the average of the real and generated flows, respectively.

The Normalized Root Mean Squared Error (NRMSE) is defined as follows:

$$\text{NRMSE} = \frac{\sqrt{\frac{1}{n}\sum_{i=1}^{n}(y_i - \hat{y}_i)^2}}{\max(y_i, \hat{y}_i) - \min(y_i, \hat{y}_i)} \tag{2}$$

where $\max y_i, \hat{y}_i$ is the maximum flow and $\min y_i, \hat{y}_i$ is the minimum flow. Lower values indicate better performance.

The Jensen-Shannon (JS) divergence is a measure to assess the similarity between two distributions. It is based on the Kullback-Leibler divergence (KLD) but it is symmetric ($JS(P||Q) = JS(Q||P)$) and ranges in $[0, 1]$. Formally, given two probability distributions P and Q, and $M = \frac{1}{2}(P||Q)$, we define the JS divergence as:

$$\text{JSD}(P||Q) = \frac{1}{2}\text{KLD}(P||M) + \frac{1}{2}\text{KLD}(Q||M). \tag{3}$$

KLD measures how different a probability distribution is from a reference probability distribution. Formally, given two discrete probability distributions P and Q, defined on the same probability

space X, the KL divergence from P to Q is defined as:

$$\text{KLD}(P||Q) = \sum_{x \in X} P(x) log\left(\frac{P(x)}{Q(x)}\right). \tag{4}$$

Formally, given two probability distributions P and Q, KLD is the expectation of the logarithmic difference between the probabilities of P and Q, where the expectation is taken using the probabilities of P. KLD is always non-negative (KLD$(P||Q) \geq 0$) and not symmetric, i.e., KLD$(P||Q) \neq$ KLD$(Q||P)$. P and Q are the same distribution if KLD$(P||Q) = 0$.

Supplementary Note 2: DG-sum and DG-Knn. Each flow in Deep Gravity is described by 39 features (18 geographic features of the origin and 18 of the destination, distance between origin and destination, and their population). We also consider a light version of Deep Gravity, namely DG-sum, in which we just count a location's total number of POIs without distinguishing among the categories (5 features per flow), and a more complex version of Deep Gravity, namely DG-Knn, which includes the geographic features of the k nearest locations to a flow's origin and destination (77 features per flow).

DG-sum considers as geographic feature of the origin (destination) just the total number of POIs regardless of the specific category they belong to. Each flow in DG-sum is hence described by 5 features only (sum of POIs, distance between origin and destination and their population). We find that DG-sum has a lower performance than DG, regardless the decile of the population considered and the dataset (England in Supplementary Figure 5a, Italy in Supplementary Figure 5c, New York State in Supplementary Figure 5 e).

These results suggest that splitting POIs in categories brings a significant contribution, especially in England where DG has an improvement of around 141.98% on DG-sum. While this improvement is less marked in Italy and New York State, we find an improvement of DG over DG-sum up to 17.90% (Italy) and 6.05% (New York State).

DG-Knn considers the geographic features of the k nearest locations (OAs, CAs, or CTs) to a flow's origin and destination. The k nearest locations to the origin (destination) are computed by sorting all locations in decreasing order with respect to the geographic distance to the origin (destination) and then selecting the top k. We then train a model that exploits the geographic features of the origin, the geographic features of the destination, and the feature-wise average of the k nearest locations to the origin and the destination. We perform experiments for $k = 2, \dots, 9$ and do not find any significant improvement of DG-Knn's performance with respect to DG, regardless of the value of k. In England, we observe an almost constant decrease of the relative improvement of DG-Knn with respect to DG, again regardless of the value of k (Supplementary Figure 4a, b). In Italy, we find a slight increase of the performances in the least populated areas and, in general, a decrease of DG-Knn's performance up to the 10% with respect to DG (Supplementary Figure 4c, d). For New York State, DG-Knn slightly improves on DG, but this improvement is in any case lower than 3% (Supplementary Figure 4e, f).

Supplementary Note 3: predicted versus observed flows. Supplementary Figures 1 and 3 show the CPC and the Pearson correlation coefficient between the observed flows and the generated flows for Middle Layer Super Output Areas (MSOAs) in England for regions of interest of 25km and

10km, respectively. MSOAs are an aggregation of adjacent OAs with similar social characteristics; they generally contain 5,000 to 15,000 residents and 2,000 to 6,000 households. Plots with a higher concentration of points closer to the main diagonal have a higher correlation and a higher CPC. Note that, being MSOAs aggregations of OAs, the overall CPC is higher than that for OAs.

For regions of interest of size 25km, DG (Supplementary Figure 1a) has an improvement over G of of 0.212 in terms of CPC (+34%) and of 0.183 in terms of Pearson correlation (+25%, Supplementary Figure 1d). DG has an improvement of 0.207 (CPC) and 0.172 (correlation) over NG (Supplementary Figure 1c) and of 0.124 (CPC) and 0.08 (correlation) over MFG (Supplementary Figure 1b).

For regions of interest of 10km, DG (Supplementary Figure 3a) has an improvement over G (Supplementary Figure 3d) of 0.131 (+19.5%) in terms of CPC and of 0.143 (+18%) in terms of correlation. DG also present an improvement of 0.125 (CPC) and of 0.128 (correlation) with respect to NG (Supplementary Figure 3c). Also, DG has an improvement over MFG (Supplementary Figure 3b) of 0.048 in terms of CPC and of 0.047 in terms of correlation. In general, with regions of interest of size 10km the improvement of DG over the other models is smaller.

Overall, DG achieves a significant improvement in the realism of the generated flows with respect to both the gravity model and models that do not use non-linearity or geographic information.

Supplementary Note 4: leave-one-city-out. In the leave-one-city-out validation we train in turn DG on all the regions of interest of eight cities and test it on the remaining one. In other words, we test the ability of DG of generating flows for a city that it "never seen", i.e., a city for which no region of interest is used during the training phase. Supplementary Figure 2 compares the results of DG with the results of Leave-one-city-out DG (L-DG) for the 9 core cities[1] in England (Leeds, Sheffield, Birmingham, Bristol, Liverpool, Manchester, Newcastle, Nottingham) and London. L-DG is remarkably close to DG, suggesting that DG is a geographic agnostic model.

Supplementary Note 5: local explanations. In Supplementary Figures 6 and 7, we show local explanation of flows for Italy and New York State, respectively. Features are reported on the vertical axis, sorted from the most relevant on top to the least relevant on the bottom. The value of the feature is indicated in gray on the left of the feature name. The bars denote the contribution of each feature to the model's prediction (the number inside or near the bar is the Shapely value). The sum of the Shapely values of all features is equal to the model's prediction ($f(x)$ denotes the score): feature with positive (negative) Shapely values push the flow probability to higher (lower) values with respect to the model's average prediction $E[f(x)]$. As confirmed in the global explanation discussed in Figure 5 of the main paper, the population and the distance play an important role in generating the flow while the other factors are barely considered (e.g., lower Shapely values).

[1]https://www.corecities.com/
