# Supplementary material for: A Deep Gravity model for mobility flows generation
Source: Nat Commun. 2021 Nov 12;12:6576. doi: 10.1038/s41467-021-26752-4 (PMC8589995; doi:10.1038/s41467-021-26752-4)
Supplement: Supplementary file 2 — Reporting Summary [file 41467_2021_26752_MOESM2_ESM.pdf]

nature portfolio

Corresponding author(s): Luca Pappalardo

Last updated by author(s): Oct 16, 2021

# Reporting Summary

Nature Portfolio wishes to improve the reproducibility of the work that we publish. This form provides structure for consistency and transparency in reporting. For further information on Nature Portfolio policies, see our Editorial Policies and the Editorial Policy Checklist.

## Statistics

For all statistical analyses, confirm that the following items are present in the figure legend, table legend, main text, or Methods section.

| n/a | Confirmed | |
|---|---|---|
| ☐ | ☒ | The exact sample size ($n$) for each experimental group/condition, given as a discrete number and unit of measurement |
| ☐ | ☒ | A statement on whether measurements were taken from distinct samples or whether the same sample was measured repeatedly |
| ☒ | ☐ | The statistical test(s) used AND whether they are one- or two-sided<br>*Only common tests should be described solely by name; describe more complex techniques in the Methods section.* |
| ☒ | ☐ | A description of all covariates tested |
| ☒ | ☐ | A description of any assumptions or corrections, such as tests of normality and adjustment for multiple comparisons |
| ☐ | ☒ | A full description of the statistical parameters including central tendency (e.g. means) or other basic estimates (e.g. regression coefficient) AND variation (e.g. standard deviation) or associated estimates of uncertainty (e.g. confidence intervals) |
| ☒ | ☐ | For null hypothesis testing, the test statistic (e.g. $F$, $t$, $r$) with confidence intervals, effect sizes, degrees of freedom and $P$ value noted<br>*Give P values as exact values whenever suitable.* |
| ☒ | ☐ | For Bayesian analysis, information on the choice of priors and Markov chain Monte Carlo settings |
| ☒ | ☐ | For hierarchical and complex designs, identification of the appropriate level for tests and full reporting of outcomes |
| ☒ | ☐ | Estimates of effect sizes (e.g. Cohen's $d$, Pearson's $r$), indicating how they were calculated |

*Our web collection on statistics for biologists contains articles on many of the points above.*

## Software and code

Policy information about availability of computer code

| | |
|---|---|
| Data collection | Commuting data are public and/or collected by official statistics institutes. Data about Points of Interest (POIs) have been downloaded from OpenStreetMap using our own code. |
| Data analysis | The analysis of the data have been performed using our own custm code developed on purpose. The code can be found at https://github.com/scikit-mobility/DeepGravity |

For manuscripts utilizing custom algorithms or software that are central to the research but not yet described in published literature, software must be made available to editors and reviewers. We strongly encourage code deposition in a community repository (e.g. GitHub). See the Nature Portfolio guidelines for submitting code & software for further information.

## Data

Policy information about availability of data

All manuscripts must include a data availability statement. This statement should provide the following information, where applicable:

- Accession codes, unique identifiers, or web links for publicly available datasets
- A description of any restrictions on data availability
- For clinical datasets or third party data, please ensure that the statement adheres to our policy

The commuting data for England are freely available at https://census.ukdataservice.ac.uk/use-data/guides/flow-data.aspx and https://census.ukdataservice.ac.uk/use-data/guides/boundary-data

The commuting data for Italy are freely available at http://datiopen.istat.it/datasetPND.php and https://www.istat.it/it/archivio/104317

The flows data in New York State are freely available at github.com/GeoDS/COVID19USFlowsand

# Field-specific reporting

Please select the one below that is the best fit for your research. If you are not sure, read the appropriate sections before making your selection.

☐ Life sciences ☒ Behavioural & social sciences ☐ Ecological, evolutionary & environmental sciences

For a reference copy of the document with all sections, see nature.com/documents/nr-reporting-summary-flat.pdf

# Behavioural & social sciences study design

All studies must disclose on these points even when the disclosure is negative.

| | |
|---|---|
| Study description | We develop the Deep Gravity model, an effective method to generate flow probabilities that exploits many quantitative features (e.g., land use, road network, transport, food, health facilities) extracted from voluntary geographic data, and uses deep neural networks to discover non-linear relationships between those features and mobility flows.<br>We perform experiments on mobility flows in England, Italy, and New York State to show that Deep Gravity has good geographic generalization capability, achieving a significant increase in performance, especially in densely populated regions of interest, with respect to the classic gravity model and models that do not use deep neural networks or geographic data. |
| Research sample | For England, we use commuting flows between Output Areas (OAs) collected by the UK census 2011, which covers 30,008,634 commuters with an average flow from an OA to another of 1.78 and a standard deviation of 3.21. The Italian census covers 15,003,287 commuters with an average flow of 2.07 and a standard deviation of 4.27.<br>Finally, in New York State, there are 41,070,279 commuters with an average of 66.86 people traveling between between Census Tracts (CTs) and a standard deviation of 364.58. |
| Sampling strategy | We use all data available from the dataset described above. |
| Data collection | Commuting data used are public and/or collected by official statistics institutes. Data about Points of Interest (POIs) have been downloaded from OpenStreetMap using our own code. |
| Timing | Commuting data for England and Italy refer to UK and Italian censuses 2011. Flows data for New York State refer to year 2019. |
| Data exclusions | No data were excluded from the analysis. |
| Non-participation | N/A, no participants other than the authors were involved in the study. |
| Randomization | N/A, no participants other than the authors were involved in the study. |

# Reporting for specific materials, systems and methods

We require information from authors about some types of materials, experimental systems and methods used in many studies. Here, indicate whether each material, system or method listed is relevant to your study. If you are not sure if a list item applies to your research, read the appropriate section before selecting a response.

## Materials & experimental systems

| n/a | Involved in the study |
|---|---|
| ☒ | ☐ Antibodies |
| ☒ | ☐ Eukaryotic cell lines |
| ☒ | ☐ Palaeontology and archaeology |
| ☒ | ☐ Animals and other organisms |
| ☒ | ☐ Human research participants |
| ☒ | ☐ Clinical data |
| ☒ | ☐ Dual use research of concern |

## Methods

| n/a | Involved in the study |
|---|---|
| ☒ | ☐ ChIP-seq |
| ☒ | ☐ Flow cytometry |
| ☒ | ☐ MRI-based neuroimaging |
